# Crystal structure of (*E*)-5-di­ethyl­amino-2-({[4-(di­methyl­amino)­phen­yl]imino}­meth­yl)phenol

**DOI:** 10.1107/S2056989015011779

**Published:** 2015-06-24

**Authors:** C. Vidya Rani, G. Chakkaravarthi, G. Rajagopal

**Affiliations:** a PG & Research Department of Chemistry, Chikkanna Government Arts College, Tiruppur 641 602, India; b Department of Physics, CPCL Polytechnic College, Chennai 600 068, India

**Keywords:** crystal structure, Schiff base, intra­molecular O—H⋯N hydrogen bond, C—H⋯π inter­actions

## Abstract

The title Schiff base compound, C_19_H_25_N_3_O, is approximately planar, with a dihedral angle of 9.03 (13)° between the planes of the aromatic rings, and has an *E* conformation about the N=C bond. The mol­ecular structure is stabilized by an intra­molecular O—H⋯N hydrogen bond, with an *S*(6) ring motif. In the crystal, mol­ecules are linked by C—H⋯π inter­actions, forming sheets parallel to the *bc* plane.

## Related literature   

For biological activities of Schiff base derivatives, see: Savaliya *et al.* (2010 ▸); Xu *et al.* (2012 ▸). For the structures of similar compounds, see: Manvizhi *et al.* (2011 ▸); Thirugnanasundar *et al.* (2011 ▸).
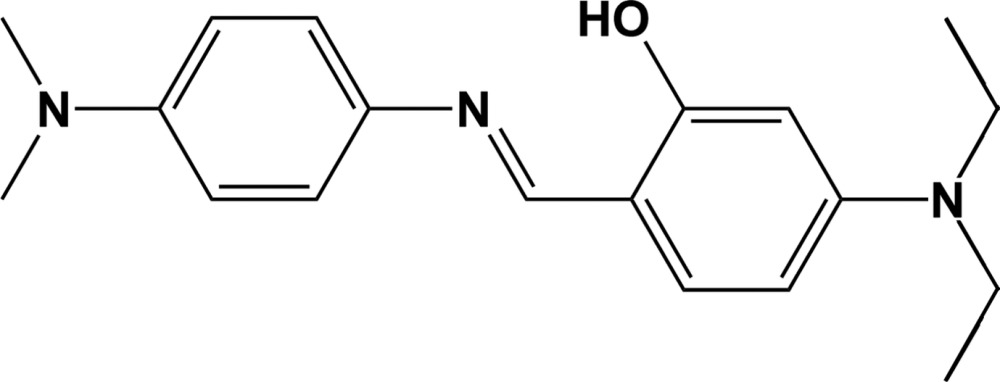


## Experimental   

### Crystal data  

C_19_H_25_N_3_O
*M* *_r_* = 311.42
Monoclinic, 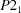

*a* = 8.8201 (7) Å
*b* = 7.8850 (7) Å
*c* = 13.0639 (10) Å
β = 108.407 (3)°
*V* = 862.06 (12) Å^3^
*Z* = 2
Mo *K*α radiation
μ = 0.08 mm^−1^
*T* = 295 K
0.26 × 0.22 × 0.20 mm

### Data collection  

Bruker Kappa APEXII CCD diffractometer
Absorption correction: multi-scan (*SADABS*; Sheldrick, 1996 ▸) *T*_min_ = 0.981, *T*_max_ = 0.985
13009 measured reflections
3825 independent reflections
2438 reflections with *I* > 2σ(*I*)
*R*_int_ = 0.027

### Refinement  

*R*[*F* ^2^ > 2σ(*F* ^2^)] = 0.052
*wR*(*F* ^2^) = 0.159
*S* = 1.03
3825 reflections
214 parameters
6 restraints
H-atom parameters constrained
Δρ_max_ = 0.29 e Å^−3^
Δρ_min_ = −0.16 e Å^−3^

### 

Data collection: *APEX2* (Bruker, 2004 ▸); cell refinement: *SAINT* (Bruker, 2004 ▸); data reduction: *SAINT*; program(s) used to solve structure: *SHELXS97* (Sheldrick, 2008 ▸); program(s) used to refine structure: *SHELXL97* (Sheldrick, 2008 ▸); molecular graphics: *PLATON* (Spek, 2009 ▸) and *Mercury* (Macrae *et al.*, 2008 ▸); software used to prepare material for publication: *SHELXL97* and *PLATON*.

## Supplementary Material

Crystal structure: contains datablock(s) global, I. DOI: 10.1107/S2056989015011779/su5157sup1.cif

Structure factors: contains datablock(s) I. DOI: 10.1107/S2056989015011779/su5157Isup2.hkl

Supporting information file. DOI: 10.1107/S2056989015011779/su5157Isup3.cml

**Fig. 1.**
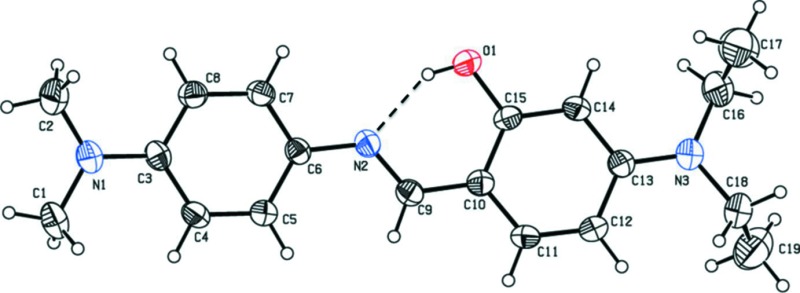
The molecular structure of the title compound, with atom labelling. Displacement ellipsoids are drawn at the 30% probability level. The intramolecular O—H..N hydrogen bonds is shown as a dashd lines (see Table 1 for details).

**Fig. 2.**
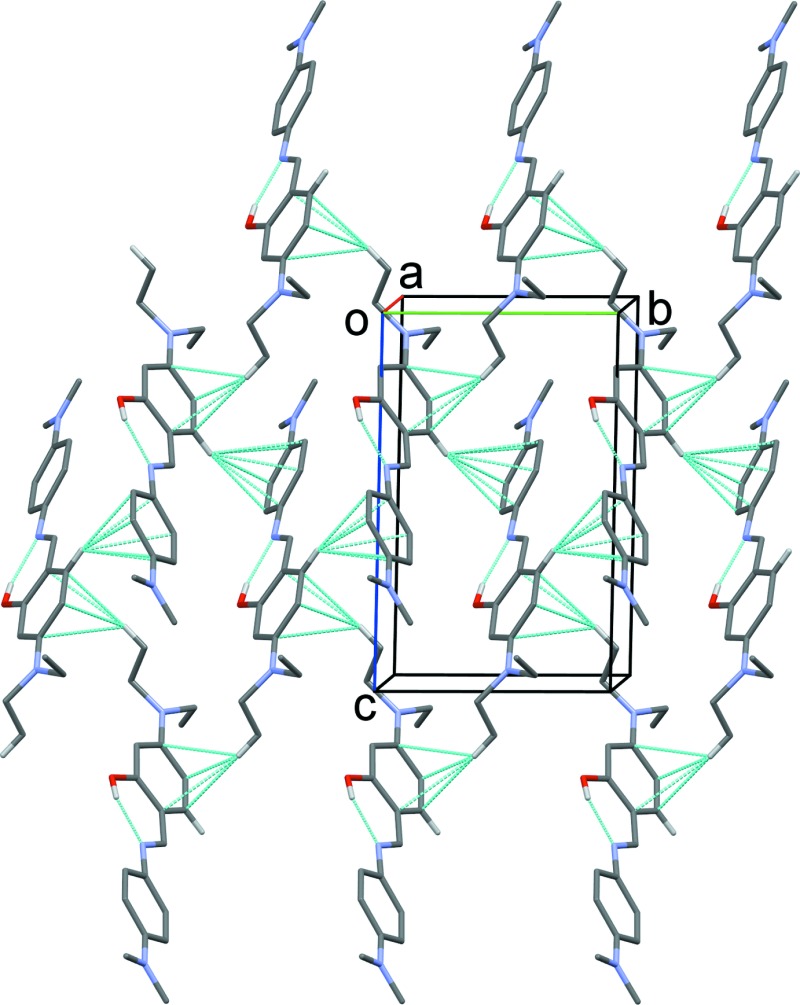
A view along the a axis of the crystal apcking of the title compound. The O—H..N and C-H···π interactions are illustrated by dashed lines (see Table 1 for details).

. DOI: 10.1107/S2056989015011779/su5157fig1.tif
The mol­ecular structure of the title compound, with atom labelling. Displacement ellipsoids are drawn at the 30% probability level. The intra­molecular O—H.·N hydrogen bonds is shown as a dashd lines (see Table 1 for details).

a . DOI: 10.1107/S2056989015011779/su5157fig2.tif
A view along the *a* axis of the crystal apcking of the title compound. The O—H.·N and C-H⋯π inter­actions are illustrated by dashed lines (see Table 1 for details).

CCDC reference: 1407678

Additional supporting information:  crystallographic information; 3D view; checkCIF report

## Figures and Tables

**Table 1:** Hydrogen-bond geometry (, ) *Cg*1 and *Cg*2 are the centroids of rings C3C8 and C10C15, respectively.

*D*H*A*	*D*H	H*A*	*D* *A*	*D*H*A*
O1H1N2	0.82	1.85	2.585(3)	148
C11H11*Cg*1^i^	0.93	2.71	3.517(3)	145
C17H17*B* *Cg*2^ii^	0.96	2.90	3.743(5)	147

Symmetry codes: (i) 
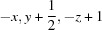
; (ii) 
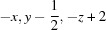
.

## supplementary crystallographic information

### S1. Structural commentary

Schiff base derivatives are known to exhibit anti­microbial (Savaliya *et al.*, 2010) and anti­bacterial (Xu *et al.*, 2012) activities. Herein we report on the synthesis and the crystal structure of a new Schiff base compound.

The molecular structure of the title compound is illustrated in Fg. 1. The geometric parameters are comparable to those reported for similar structures (Manvizhi *et al.*, 2011; Thirugnanasundar *et al.*, 2011). The dihedral angle between the benzene rings (C3—C8) and (C10—C15) is 9.03 (13)°. The molecular structure is stabilized by an intra­molecular O—H···N hydrogen bond (Table 1 and Fig. 1).

In the crystal, molecules are linked by C—H···π inter­actions forming sheets parallel to the bc plane (Table 1 and Fig. 2).

### S2. Synthesis and crystallization

To an ethanol solution (10 ml) of 5-(di­ethyl­amino)-2-hy­droxy­benzaldehyde (96.5 mg, 0.5 mol) was added *N^1^,N^1^*-di­methyl­benzene-1,4-di­amine (68 mg, 0.5 mol). The mixture was stirred and 2 to 3 drops of glacial acetic acid were added. Stirring was continued for 30 mins and then the reaction mixture was refluxed for 2 h. On completion of the reaction, monitored by TLC, the mixture was allowed to cool to room temperature and the solid yellow precipitate that formed was filtered, dried, and recrystallized from DMF, giving colourless block-like crystals.

### S3. Refinement

Crystal data, data collection and structure refinement details are summarized in Table 2. H atoms were positioned geometrically and refined using riding model: O—H = 0.82 Å, C—H = 0.93 - 0.97 Å with *U*_iso_(H) = 1.5Ueq(O,C) for the hydroxyl and methyl H atoms and 1.2U_eq_(C) for other H atoms. The components of the anisotropic displacement parameters of the atoms in bonds N3—C16, N3—C18 and N1—C2 were restrained to be equal within an effective standard deviation of 0.001 using the DELU command, and the C16—C17 bond distance was restrained to 1.54 (1) Å.

### Crystal data

**Table:**

C_19_H_25_N_3_O	*F*(000) = 336
*M**_r_* = 311.42	*D*_x_ = 1.200 Mg m^−^^3^
Monoclinic, *P*2_1_	Mo *K*α radiation, λ = 0.71073 Å
Hall symbol: P 2yb	Cell parameters from 4428 reflections
*a* = 8.8201 (7) Å	θ = 2.4–27.2°
*b* = 7.8850 (7) Å	µ = 0.08 mm^−^^1^
*c* = 13.0639 (10) Å	*T* = 295 K
β = 108.407 (3)°	Block, colourless
*V* = 862.06 (12) Å^3^	0.26 × 0.22 × 0.20 mm
*Z* = 2	

### Data collection

**Table:**

Bruker Kappa APEXII CCD diffractometer	3825 independent reflections
Radiation source: fine-focus sealed tube	2438 reflections with *I* > 2σ(*I*)
Graphite monochromator	*R*_int_ = 0.027
ω and φ scan	θ_max_ = 27.3°, θ_min_ = 2.4°
Absorption correction: multi-scan (*SADABS*; Sheldrick, 1996)	*h* = −11→10
*T*_min_ = 0.981, *T*_max_ = 0.985	*k* = −10→10
13009 measured reflections	*l* = −16→16

### Refinement

**Table:**

Refinement on *F*^2^	Secondary atom site location: difference Fourier map
Least-squares matrix: full	Hydrogen site location: inferred from neighbouring sites
*R*[*F*^2^ > 2σ(*F*^2^)] = 0.052	H-atom parameters constrained
*wR*(*F*^2^) = 0.159	*w* = 1/[σ^2^(*F*_o_^2^) + (0.0751*P*)^2^ + 0.1538*P*] where *P* = (*F*_o_^2^ + 2*F*_c_^2^)/3
*S* = 1.03	(Δ/σ)_max_ < 0.001
3825 reflections	Δρ_max_ = 0.29 e Å^−^^3^
214 parameters	Δρ_min_ = −0.16 e Å^−^^3^
6 restraints	Extinction correction: *SHELXL97* (Sheldrick, 2008), Fc^*^=kFc[1+0.001xFc^2^λ^3^/sin(2θ)]^-1/4^
Primary atom site location: structure-invariant direct methods	Extinction coefficient: 0.014 (4)

### Special details

**Table:**

Geometry. All esds (except the esd in the dihedral angle between two l.s. planes) are estimated using the full covariance matrix. The cell esds are taken into account individually in the estimation of esds in distances, angles and torsion angles; correlations between esds in cell parameters are only used when they are defined by crystal symmetry. An approximate (isotropic) treatment of cell esds is used for estimating esds involving l.s. planes.
Refinement. Refinement of F^2^ against ALL reflections. The weighted R-factor wR and goodness of fit S are based on F^2^, conventional R-factors R are based on F, with F set to zero for negative F^2^. The threshold expression of F^2^ > 2sigma(F^2^) is used only for calculating R-factors(gt) etc. and is not relevant to the choice of reflections for refinement. R-factors based on F^2^ are statistically about twice as large as those based on F, and R- factors based on ALL data will be even larger.

### Fractional atomic coordinates and isotropic or equivalent isotropic displacement parameters (Å^2^)

**Table:**

	*x*	*y*	*z*	*U*_iso_*/*U*_eq_	
C1	0.4431 (4)	0.6814 (5)	0.2015 (2)	0.0780 (10)	
H1A	0.3484	0.6253	0.1573	0.117*	
H1B	0.5213	0.6840	0.1644	0.117*	
H1C	0.4171	0.7953	0.2159	0.117*	
C2	0.6641 (4)	0.5264 (6)	0.3287 (3)	0.0902 (12)	
H2A	0.7316	0.5867	0.3901	0.135*	
H2B	0.7039	0.5405	0.2688	0.135*	
H2C	0.6635	0.4081	0.3459	0.135*	
C3	0.4220 (3)	0.5850 (3)	0.3736 (2)	0.0502 (6)	
C4	0.2713 (3)	0.6598 (4)	0.3525 (2)	0.0547 (7)	
H4	0.2275	0.7180	0.2880	0.066*	
C5	0.1861 (3)	0.6502 (4)	0.42371 (19)	0.0512 (6)	
H5	0.0860	0.7008	0.4067	0.061*	
C6	0.2487 (3)	0.5648 (3)	0.52175 (18)	0.0444 (6)	
C7	0.3965 (3)	0.4922 (3)	0.5425 (2)	0.0508 (6)	
H7	0.4405	0.4352	0.6075	0.061*	
C8	0.4820 (3)	0.5003 (4)	0.4715 (2)	0.0537 (7)	
H8	0.5816	0.4483	0.4889	0.064*	
C9	0.0435 (3)	0.6249 (4)	0.59847 (19)	0.0481 (6)	
H9	−0.0034	0.6975	0.5411	0.058*	
C10	−0.0294 (3)	0.6066 (3)	0.68154 (19)	0.0462 (6)	
C11	−0.1669 (3)	0.6947 (3)	0.6784 (2)	0.0537 (7)	
H11	−0.2150	0.7626	0.6188	0.064*	
C12	−0.2347 (3)	0.6864 (4)	0.7586 (2)	0.0596 (8)	
H12	−0.3278	0.7467	0.7524	0.072*	
C13	−0.1648 (3)	0.5871 (4)	0.8507 (2)	0.0528 (6)	
C14	−0.0279 (3)	0.4940 (3)	0.8547 (2)	0.0523 (7)	
H14	0.0198	0.4255	0.9141	0.063*	
C15	0.0370 (3)	0.5025 (3)	0.7722 (2)	0.0472 (6)	
C16	−0.1784 (4)	0.4511 (5)	1.0194 (3)	0.0834 (10)	
H16A	−0.2702	0.4176	1.0403	0.100*	
H16B	−0.1393	0.3520	0.9915	0.100*	
C17	−0.0541 (5)	0.5166 (6)	1.1125 (3)	0.1089 (14)	
H17A	0.0389	0.5432	1.0925	0.163*	
H17B	−0.0274	0.4327	1.1686	0.163*	
H17C	−0.0917	0.6173	1.1380	0.163*	
C18	−0.3507 (4)	0.7080 (5)	0.9409 (3)	0.0835 (10)	
H18A	−0.3375	0.7326	1.0160	0.100*	
H18B	−0.3372	0.8131	0.9062	0.100*	
C19	−0.5106 (4)	0.6420 (8)	0.8890 (4)	0.1178 (15)	
H19A	−0.5223	0.6129	0.8156	0.177*	
H19B	−0.5881	0.7267	0.8905	0.177*	
H19C	−0.5270	0.5428	0.9268	0.177*	
N1	0.5063 (3)	0.5915 (4)	0.30117 (18)	0.0693 (7)	
N2	0.1707 (2)	0.5451 (3)	0.60028 (16)	0.0495 (5)	
N3	−0.2261 (3)	0.5836 (4)	0.9344 (2)	0.0826 (9)	
O1	0.1678 (2)	0.4075 (3)	0.77919 (17)	0.0695 (6)	
H1	0.2010	0.4308	0.7289	0.104*	

### Atomic displacement parameters (Å^2^)

**Table:**

	*U*^11^	*U*^22^	*U*^33^	*U*^12^	*U*^13^	*U*^23^
C1	0.097 (2)	0.090 (3)	0.0568 (17)	−0.0042 (19)	0.0384 (16)	0.0063 (17)
C2	0.0743 (17)	0.127 (3)	0.082 (2)	0.0100 (19)	0.0428 (18)	0.012 (2)
C3	0.0561 (14)	0.0511 (15)	0.0454 (13)	−0.0092 (13)	0.0189 (11)	0.0004 (12)
C4	0.0608 (16)	0.0587 (17)	0.0431 (13)	0.0034 (14)	0.0143 (12)	0.0113 (13)
C5	0.0487 (13)	0.0557 (16)	0.0487 (14)	0.0064 (12)	0.0148 (11)	0.0060 (13)
C6	0.0510 (14)	0.0410 (14)	0.0418 (13)	−0.0028 (12)	0.0154 (11)	0.0013 (11)
C7	0.0506 (14)	0.0535 (16)	0.0453 (14)	0.0033 (13)	0.0110 (11)	0.0073 (12)
C8	0.0494 (14)	0.0554 (16)	0.0544 (16)	0.0018 (12)	0.0137 (12)	0.0038 (13)
C9	0.0505 (14)	0.0473 (15)	0.0436 (13)	−0.0018 (13)	0.0107 (11)	0.0001 (11)
C10	0.0455 (13)	0.0444 (14)	0.0467 (13)	−0.0009 (11)	0.0116 (10)	−0.0012 (11)
C11	0.0547 (14)	0.0533 (16)	0.0496 (14)	0.0138 (12)	0.0113 (12)	0.0110 (12)
C12	0.0529 (14)	0.066 (2)	0.0604 (16)	0.0188 (13)	0.0188 (13)	0.0095 (14)
C13	0.0533 (14)	0.0548 (16)	0.0530 (14)	0.0039 (13)	0.0207 (11)	0.0075 (13)
C14	0.0543 (15)	0.0523 (16)	0.0506 (15)	0.0101 (13)	0.0167 (12)	0.0147 (13)
C15	0.0403 (12)	0.0443 (14)	0.0554 (15)	0.0053 (11)	0.0129 (11)	0.0050 (12)
C16	0.086 (2)	0.093 (3)	0.081 (2)	0.0042 (18)	0.0414 (19)	0.0168 (16)
C17	0.126 (3)	0.098 (3)	0.097 (3)	0.010 (3)	0.028 (3)	−0.008 (3)
C18	0.080 (2)	0.107 (3)	0.075 (2)	0.0190 (17)	0.0400 (18)	0.0039 (19)
C19	0.094 (3)	0.134 (4)	0.131 (4)	0.014 (3)	0.043 (3)	−0.015 (3)
N1	0.0737 (14)	0.083 (2)	0.0611 (15)	0.0044 (13)	0.0357 (13)	0.0146 (14)
N2	0.0530 (12)	0.0515 (14)	0.0459 (11)	0.0007 (10)	0.0184 (9)	0.0030 (10)
N3	0.0769 (16)	0.112 (2)	0.0696 (16)	0.0288 (15)	0.0381 (13)	0.0210 (15)
O1	0.0666 (12)	0.0777 (14)	0.0737 (14)	0.0302 (11)	0.0356 (10)	0.0283 (11)

### Geometric parameters (Å, º)

**Table:**

C1—N1	1.432 (4)	C11—C12	1.363 (4)
C1—H1A	0.9600	C11—H11	0.9300
C1—H1B	0.9600	C12—C13	1.404 (4)
C1—H1C	0.9600	C12—H12	0.9300
C2—N1	1.419 (4)	C13—N3	1.364 (3)
C2—H2A	0.9600	C13—C14	1.400 (3)
C2—H2B	0.9600	C14—C15	1.373 (4)
C2—H2C	0.9600	C14—H14	0.9300
C3—N1	1.377 (3)	C15—O1	1.354 (3)
C3—C8	1.390 (4)	C16—C17	1.451 (5)
C3—C4	1.400 (4)	C16—N3	1.485 (4)
C4—C5	1.370 (4)	C16—H16A	0.9700
C4—H4	0.9300	C16—H16B	0.9700
C5—C6	1.397 (3)	C17—H17A	0.9600
C5—H5	0.9300	C17—H17B	0.9600
C6—C7	1.370 (3)	C17—H17C	0.9600
C6—N2	1.413 (3)	C18—C19	1.454 (5)
C7—C8	1.370 (4)	C18—N3	1.495 (4)
C7—H7	0.9300	C18—H18A	0.9700
C8—H8	0.9300	C18—H18B	0.9700
C9—N2	1.280 (3)	C19—H19A	0.9600
C9—C10	1.433 (3)	C19—H19B	0.9600
C9—H9	0.9300	C19—H19C	0.9600
C10—C11	1.386 (3)	O1—H1	0.8200
C10—C15	1.407 (3)		
			
N1—C1—H1A	109.5	N3—C13—C14	121.0 (2)
N1—C1—H1B	109.5	N3—C13—C12	121.3 (2)
H1A—C1—H1B	109.5	C14—C13—C12	117.7 (2)
N1—C1—H1C	109.5	C15—C14—C13	120.9 (2)
H1A—C1—H1C	109.5	C15—C14—H14	119.5
H1B—C1—H1C	109.5	C13—C14—H14	119.5
N1—C2—H2A	109.5	O1—C15—C14	118.5 (2)
N1—C2—H2B	109.5	O1—C15—C10	119.9 (2)
H2A—C2—H2B	109.5	C14—C15—C10	121.6 (2)
N1—C2—H2C	109.5	C17—C16—N3	109.7 (3)
H2A—C2—H2C	109.5	C17—C16—H16A	109.7
H2B—C2—H2C	109.5	N3—C16—H16A	109.7
N1—C3—C8	121.2 (2)	C17—C16—H16B	109.7
N1—C3—C4	122.3 (2)	N3—C16—H16B	109.7
C8—C3—C4	116.4 (2)	H16A—C16—H16B	108.2
C5—C4—C3	122.2 (2)	C16—C17—H17A	109.5
C5—C4—H4	118.9	C16—C17—H17B	109.5
C3—C4—H4	118.9	H17A—C17—H17B	109.5
C4—C5—C6	120.4 (2)	C16—C17—H17C	109.5
C4—C5—H5	119.8	H17A—C17—H17C	109.5
C6—C5—H5	119.8	H17B—C17—H17C	109.5
C7—C6—C5	117.3 (2)	C19—C18—N3	111.1 (3)
C7—C6—N2	117.5 (2)	C19—C18—H18A	109.4
C5—C6—N2	125.2 (2)	N3—C18—H18A	109.4
C8—C7—C6	122.6 (2)	C19—C18—H18B	109.4
C8—C7—H7	118.7	N3—C18—H18B	109.4
C6—C7—H7	118.7	H18A—C18—H18B	108.0
C7—C8—C3	121.0 (2)	C18—C19—H19A	109.5
C7—C8—H8	119.5	C18—C19—H19B	109.5
C3—C8—H8	119.5	H19A—C19—H19B	109.5
N2—C9—C10	122.5 (2)	C18—C19—H19C	109.5
N2—C9—H9	118.8	H19A—C19—H19C	109.5
C10—C9—H9	118.8	H19B—C19—H19C	109.5
C11—C10—C15	116.4 (2)	C3—N1—C2	120.8 (2)
C11—C10—C9	121.6 (2)	C3—N1—C1	120.2 (2)
C15—C10—C9	121.9 (2)	C2—N1—C1	118.6 (2)
C12—C11—C10	123.1 (2)	C9—N2—C6	123.9 (2)
C12—C11—H11	118.5	C13—N3—C16	121.5 (3)
C10—C11—H11	118.5	C13—N3—C18	121.1 (3)
C11—C12—C13	120.3 (2)	C16—N3—C18	117.4 (2)
C11—C12—H12	119.8	C15—O1—H1	109.5
C13—C12—H12	119.8		
			
N1—C3—C4—C5	178.6 (3)	C13—C14—C15—C10	1.1 (4)
C8—C3—C4—C5	−0.3 (4)	C11—C10—C15—O1	177.5 (3)
C3—C4—C5—C6	0.5 (4)	C9—C10—C15—O1	−4.4 (4)
C4—C5—C6—C7	−0.2 (4)	C11—C10—C15—C14	−2.3 (4)
C4—C5—C6—N2	−179.1 (3)	C9—C10—C15—C14	175.8 (3)
C5—C6—C7—C8	−0.3 (4)	C8—C3—N1—C2	−6.4 (5)
N2—C6—C7—C8	178.7 (2)	C4—C3—N1—C2	174.8 (3)
C6—C7—C8—C3	0.5 (4)	C8—C3—N1—C1	−179.2 (3)
N1—C3—C8—C7	−179.0 (3)	C4—C3—N1—C1	2.1 (4)
C4—C3—C8—C7	−0.2 (4)	C10—C9—N2—C6	−177.7 (2)
N2—C9—C10—C11	179.7 (2)	C7—C6—N2—C9	170.0 (2)
N2—C9—C10—C15	1.7 (4)	C5—C6—N2—C9	−11.0 (4)
C15—C10—C11—C12	1.4 (4)	C14—C13—N3—C16	−15.8 (5)
C9—C10—C11—C12	−176.7 (3)	C12—C13—N3—C16	165.5 (3)
C10—C11—C12—C13	0.8 (4)	C14—C13—N3—C18	167.5 (3)
C11—C12—C13—N3	176.7 (3)	C12—C13—N3—C18	−11.3 (5)
C11—C12—C13—C14	−2.1 (4)	C17—C16—N3—C13	97.4 (4)
N3—C13—C14—C15	−177.6 (3)	C17—C16—N3—C18	−85.7 (4)
C12—C13—C14—C15	1.2 (4)	C19—C18—N3—C13	91.4 (4)
C13—C14—C15—O1	−178.8 (3)	C19—C18—N3—C16	−85.5 (4)

### Hydrogen-bond geometry (Å, º)

Cg1 and Cg2 are the centroids of rings C3-C8 and C10-C15, respectively.

**Table:**

*D*—H···*A*	*D*—H	H···*A*	*D*···*A*	*D*—H···*A*
O1—H1···N2	0.82	1.85	2.585 (3)	148
C11—H11···*Cg*1^i^	0.93	2.71	3.517 (3)	145
C17—H17*B*···*Cg*2^ii^	0.96	2.90	3.743 (5)	147

Symmetry codes: (i) −*x*, *y*+1/2, −*z*+1; (ii) −*x*, *y*−1/2, −*z*+2.
