# Supplementary figures and images for: Crystal structure of (E)-5-di­ethyl­amino-2-({[4-(di­methyl­amino)­phen­yl]imino}­meth­yl)phenol
Source: Acta Crystallogr E Crystallogr Commun. 2015 Jun 24;71(Pt 7):o503. doi: 10.1107/S2056989015011779 (PMC4518928; doi:10.1107/S2056989015011779)

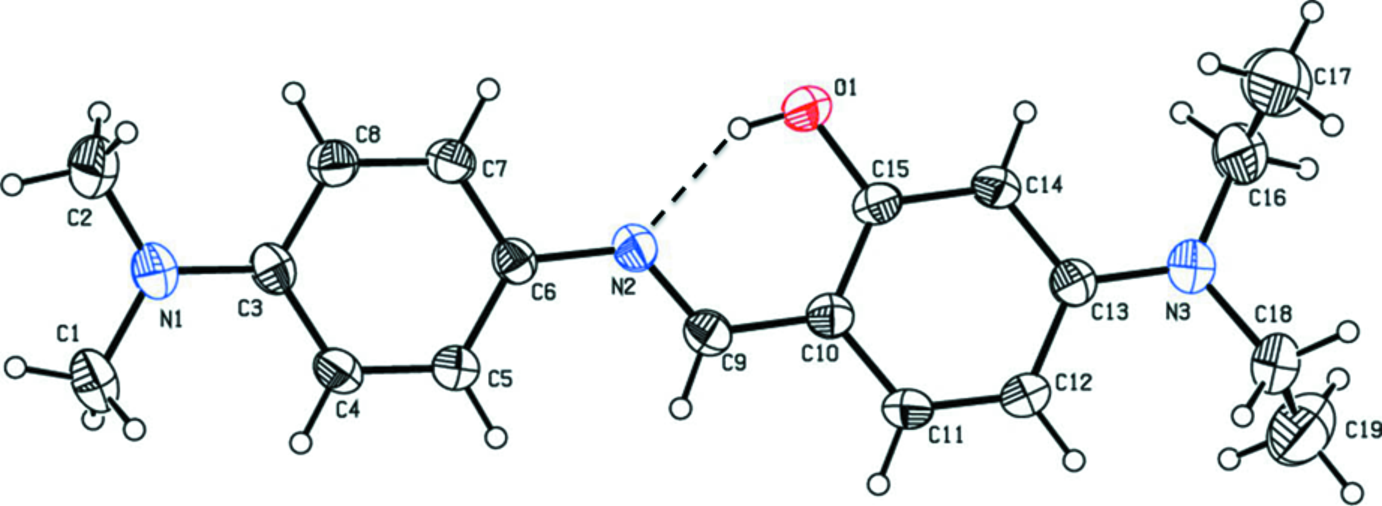

Supplement: Supplementary file 4 [file e-71-0o503-fig1.tif]

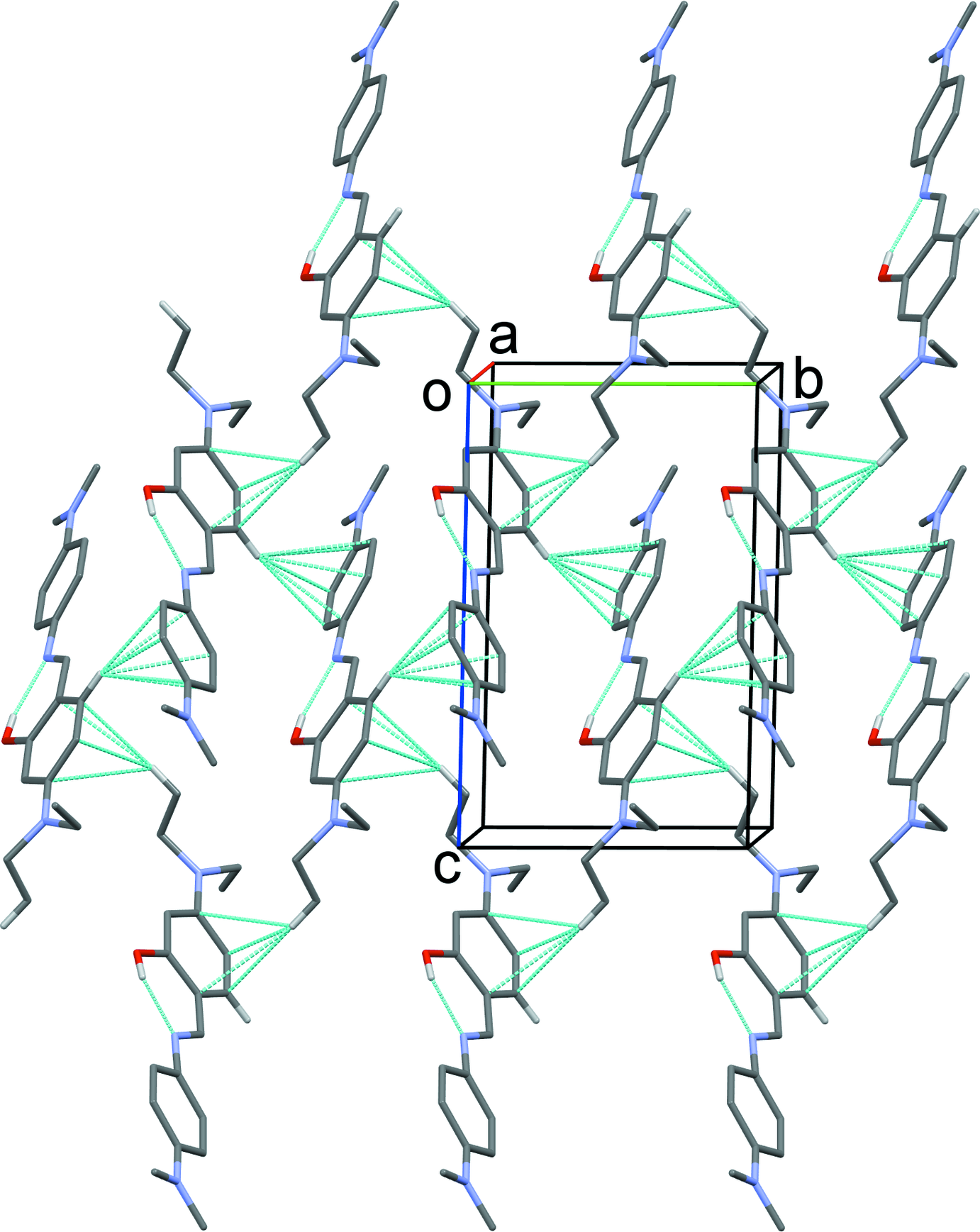

Supplement: Supplementary file 5 [file e-71-0o503-fig2.tif]
